# Understanding Value-Based Healthcare: Qualitative Insights From Nurses and Nursing Managers in Hospital Settings

**DOI:** 10.1155/jonm/9365941

**Published:** 2025-10-23

**Authors:** Carolina E. Watson, Juan M. Leyva-Moral, Nina Granel, Maria Garro, Laura Navarrete-Reyes, Rebeca Gómez-Ibáñez

**Affiliations:** ^1^Nursing Department, Faculty of Medicine, Universitat Autònoma de Barcelona, Barcelona, Spain; ^2^Centre d'Atenció Primària Sanllehy, Gerència d'Àmbit d'Atenció Primària Barcelona Ciutat, Institut Català de la Salut, Barcelona, Spain

## Abstract

**Aim:**

To explore nurses' and nursing managers' perceptions regarding Value-Based Healthcare (VBHC) and the implementation of patient-reported measures in hospital settings in Barcelona, Spain.

**Background:**

VBHC aims to improve patient outcomes while optimizing resource allocation. Patient-reported measures are essential components of this approach, yet their implementation in clinical practice remains challenging. Understanding nurses' perspectives is crucial for successful integration of VBHC principles into nursing practice.

**Methods:**

A qualitative thematic analysis approach was used. Semistructured interviews were conducted with a total of 19 participants (nurses and nursing managers) from tertiary public hospitals in Barcelona between December 2022 and June 2023. Data were analyzed using Braun and Clarke's six-phase thematic analysis framework. The study adhered to the SRQR guidelines for reporting qualitative research.

**Results:**

Four themes were identified: key elements of value in healthcare, conceptualization of VBHC through established healthcare models, practical aspects of implementing VBHC, and opportunities and systemic challenges in VBHC.

**Conclusions:**

Nurses and nursing managers in hospital settings showed a good understanding of VBHC principles, often conceptualizing them through familiar patient-centered care models. They acknowledged VBHC's potential to enhance care quality and patient outcomes but identified significant implementation challenges such as unclear strategies, need for interdisciplinary collaboration, and comprehensive training. Nurses focused mainly on personal and allocative value, with less emphasis on cost considerations and societal value.

**Implications for Nursing Management:**

Nursing managers are crucial to implementing VBHC by fostering a culture that prioritizes outcomes that matter to patients over traditional volume-based metrics. Addressing identified practical challenges and broadening the understanding of VBHC components are key steps for nurses to effectively contribute to VBHC implementation, enhance their professional recognition, and improve healthcare outcomes and value.

## 1. Introduction

Value-based healthcare (VBHC) has garnered significant attention in modern healthcare delivery, aiming to enhance patient outcomes while optimizing resource allocation and cost-effectiveness. VBHC initiatives prioritize improving healthcare quality by focusing on outcomes that matter most to patients [1].

The theoretical foundation of the concept extends beyond the simplistic metric of results per cost. Kaplan et al. [2] emphasize that VBHC should encompass multiple dimensions of value: the distribution of resources to the population (allocative value), the appropriateness of resource use for specific health needs (technical value), and the alignment of health outcomes with patient expectations (personal value). Moreover, the expert panel on effective ways of investing in health (EXPH) highlighted a fourth dimension, societal value, which includes enhancing social participation and connectivity, and is grounded in principles of solidarity and respect for diversity [2, 3]. This comprehensive definition of value in healthcare provides a framework for understanding and implementing VBHC initiatives that truly benefit patients, healthcare systems, and society. Recent analyses continue to highlight that there is no universal consensus on how VBHC should be defined and operationalized in practice, and conceptual ambiguities remain a barrier to implementation [4].

One of the essential components of this approach is the incorporation of patient-reported outcomes (PROs) and their corresponding measures such as patient-reported outcome measures (PROMs), which provide valuable insights into patients' experiences, preferences, and outcomes [5]. PROMs serve as a cornerstone of outcomes research, capturing subjective experiences, symptoms, functional status, and overall quality of life beyond traditional clinical indicators. Numerous studies have explored feasibility, acceptability, and specific experiences related to the implementation of PROMs across various clinical conditions. For instance, Short et al. [6] demonstrated the benefits of implementing same-day, self-administered electronic PROs assessment in routine HIV care, fostering improved patient–provider communication and identification of complex health issues. Current challenges include effectively translating PROMs data into actionable insights for healthcare professionals and determining optimal strategies for monitoring and incorporating PROs into daily practice. PROMs facilitate a patient-centered approach to care, enriching the understanding of treatment effectiveness and quality of life. Regarding experience, patient-reported experience measures (PREMs) also play a critical role in VBHC by focusing on patients' experiences with the care process itself, such as communication, respect, and comfort [7]. These tools—PROMs and PREMs—are collectively referred to as patient-reported measures (PRMs) and serve as the foundation of outcomes and experience research, contributing to a more holistic view of patient-centered care.

In the literature, there are examples of how to implement VBHC initiatives, considering factors such as identifying gaps, barriers, and facilitators. In this vein, Cossio-Gil et al. [8] describe how to prepare hospitals for VBHC implementation, focusing on identifying key components and organizational enablers to overcome barriers and ensure sustainability in delivering high-value care, all while adopting a patient-centered approach. In this line, May et al. [9] underscore the importance of preparing nursing leaders for VBHC through a transformative toolkit, emphasizing the need to equip healthcare professionals with the tools and knowledge necessary to succeed in delivering high-value care. Similarly, Park [10] has proposed a theory-driven implementation strategy focused on optimizing nurse staffing by balancing care quality, economic considerations, and workforce policies, with the aim of creating shared value among patients, nurses, and healthcare organizations—an approach to nursing management that shares common goals with value-based care principles. As highlighted by Zhao et al. [11], health leadership not only focuses on skill development but also emphasizes the consequence of health, which is conducive to the growth and well-being of nurses and further improves patient satisfaction—reinforcing the alignment between nursing health leadership and the core values of VBHC.

In the shift towards VBHC, nurses emerge as important agents driving innovation and patient-centered care delivery. Advanced nursing practice has been related to an improvement in patient outcomes, and the nurse role has been recognized as valuable in providing a beneficial contribution, filling up a gap in healthcare services [12]. The role of advanced practice nurses in VBHC and PROMs implementation has been explored in the literature; McCaffrey and Reinoso [13] discussed the potential of transformational leadership methods employed by advanced holistic nurses to place the patient at the center of healthcare decision-making and enhance holistic care delivery. Kleinpell et al. [14] highlight the success of national collaborations in engaging Advanced Practice Registered Nurse (APRN) teams to focus on high-value care and evidence-based practices in clinical care, though they also note that more research is needed on the specific contributions of APRNs in this context. Gálvez et al. [15] showcase innovative organizational models led by advanced practice nurses, such as the Advanced Practice Nurse in Ostomy care (APN-O) model, which demonstrates tangible improvements in healthcare outcomes. These models not only enhance the efficiency of care delivery but also optimize resource allocation, in line with VBHC principles.

However, despite the growing interest in this model and the increasing body of nursing literature exploring roles and organizational models, research specifically focusing on the subjective experiences and perceptions of nurses regarding its implementation remains limited. Most studies have focused on conceptualizing and implementing VBHC in hospital settings [16] or examining the process of implementing VBHC as an innovation [17] with particular attention to the perception of professionals regarding the implementation of self-reported measures and highlighting facilitators and barriers to their integration [18]. While there is some research on healthcare professionals' roles in VBHC, such as Van Engen et al. [19] systematic review, which identified job demands, resources, and well-being factors linked to VBHC, this led to focus on behavioral changes and job strain rather than how professionals perceive VBHC itself or its impact on their daily work. Thus, there remains a gap in understanding how healthcare professionals experience and interpret VBHC in practice, which is essential for effectively integrating nursing perspectives into policy and care delivery.

Understanding this process in specific local contexts where VBHC is being actively promoted but not yet fully established can provide valuable insights into the practical challenges and opportunities of its implementation across diverse health policy environments. While international literature has explored high-value care, this perspective has only recently begun to gain traction in our context. The importance of moving towards VBHC is increasingly recognized at the national level, with recent calls to accelerate VBHC adoption in Spain [20]. Moreover, there is a growing commitment and interest in Catalonia to transition towards a VBHC model [21, 22]. In line, the strategic plan for patient quality and safety 2023–2027 includes initiatives aimed at promoting the use of PREMs and PROMs to enhance patient experience and quality of care and foster value [23].

There are ongoing initiatives to implement PRMs and significant efforts to develop tools for electronic delivery and tracking of these measures, but the perceptions of future users, including healthcare personnel, managers, and patients, are not yet fully understood. The authors in [22] emphasize the necessity of achieving consensus on what VBHC means, including what “value” signifies for each system agent, and how to align and organize related future initiatives. Clarifying these aspects within the nursing field and aligning future initiatives accordingly will help create a joint and consensual guide to outline the path forward. Therefore, this study aims to explore nurses' and nurse managers' perceptions regarding value in healthcare, the implementation of VBHC initiatives, and PRMs delivery in hospital settings in Barcelona, Spain.

## 2. Methods

### 2.1. Design

An interpretative qualitative study with an exploratory approach has been carried out to explore the perceptions of nurses and nurse managers regarding the implementation of VBHC and outcomes measurement in hospital settings.

This interpretative methodological approach facilitates understanding individual perspectives, their beliefs, values, and meanings associated with their actions, to assess how these perspectives are integrated into their lives. In this way, it can be studied how participants construct meaning and make sense of VBHC in their clinical practice contexts. An exploratory design allows researchers to obtain comprehensive and detailed insights into participants' perspectives and identify pivotal themes without being constrained by priori categories. It is especially beneficial to build knowledge based on experience and interpretation of a phenomenon that is not well understood or has been little research. It was deemed an effective method for uncovering nuanced perspectives and contextual factors that may influence the implementation of novel healthcare models like VBHC [24, 25].

### 2.2. Participants and Setting

Participants were selected using purposive sampling to ensure a diverse range of nursing roles and positions within tertiary public hospitals in Barcelona [26]. Specifically, participants were recruited from leading tertiary care centers within the Catalonian public healthcare system. These hospitals were selected for their early adoption of VBHC in initiatives across several clinical conditions and have been recognized with national Top Value Awards [27] for their pioneering role in advancing this model in Spain. These institutions utilize PRMs in both paper and electronic formats, in some conditions integrated into their Electronic Health Records (EHR) systems to monitor patient outcomes. This sampling approach aimed at including individuals with varied experiences and perspectives related to VBHC and outcomes measurement. Nurses from various healthcare settings within the hospital environment were included. This included direct patient care nurses on hospital wards, nurses in outpatient clinics, advanced practice nurses, nursing managers, and coordinators with a nursing background.

Inclusion criteria required participants to communicate in either Spanish or Catalan, have a minimum of 3 years of clinical experience, and demonstrate familiarity with the VBHC model and PRMs. Familiarity was assessed during recruitment through a brief screening question asking participants whether they had knowledge of, or experience with, VBHC implementation and PRMs initiatives in their workplace. Exclusion criteria included nursing professionals currently on leave.

Recruitment was conducted via personal contacts within the hospitals and institutional email invitations, complemented by promotional posts shared on LinkedIn resembling poster-style invitations to raise awareness of the study. Purposive sampling was supported by nurse managers who helped identify and direct invitations to specific profiles, to ensure relevant and diverse participant inclusion. Data collection continued until data saturation was achieved, following the criteria outlined by Sandelowski [28], and it was reached at 19 participants.

### 2.3. Data Collection

Individual semistructured interviews were conducted between December 2022 and June 2023, with a total of 19 participants. Most interviews (15) were conducted face-to-face in university or hospital offices, while the remaining 4 took place online. Both formats yielded rich narratives, and during coding we did not identify format-related patterns in code application. Each interview lasted an average of 45–55 min, was audiorecorded, and transcribed verbatim.

The interview guide, which followed a predefined set of open-ended questions (see Table 1), was developed collaboratively by Carolina E. Watson and Maria Garro, with input from an expert in VBHC and qualitative methodology. It was pilot tested with two nurses outside the study sample to ensure clarity and relevance, resulting in minor adjustments. The guide comprised open-ended questions designed to facilitate in-depth exploration of the participants' perceptions and experiences. Probing questions were used as needed to elicit further details or clarify ambiguous responses.

The interviews were conducted by Carolina E. Watson, a trained qualitative researcher with a nursing background and experience in VBHC research. Carolina E. Watson did not have any formal contractual, employment, or supervisory relationship with the hospitals or participants, which fostered an open and unbiased interview environment. Reflective notes were taken during and after the interviews to capture additional insights.

### 2.4. Ethical Considerations

This research obtained approval from the Research Ethics Committee for Animal and Human Experimentation (CEEAH) of the Universitat Autònoma de Barcelona (Num. 6179). Prior to participation, all participants were assured of the confidentiality and voluntary nature of their involvement, emphasizing their right to withdraw at any point without providing a reason or facing consequences. Each study participant formally expressed consent by signing a written agreement. To safeguard anonymity, the transcribed material underwent coding and deidentification processes. The study adhered to the SRQR guidelines for reporting qualitative research [29].

### 2.5. Data Analysis

The interview data were analyzed using Braun & Clarke's [30] six-phase thematic analysis framework, conducted collaboratively by two experienced female researchers (Carolina E. Watson & Rebeca Gómez-Ibáñez) in two different phases. A separate initial review was developed to become familiar with each interview. This action allowed familiarization with the data through repeated reading of transcripts. A joint in-depth review was then conducted to identify particularities and nuances in individual experiences. Following the framework phases, initial codes were generated across the entire dataset; codes were collated into potential themes; themes were reviewed in relation to the coded extracts and the dataset, defined and named to refine their specificity, and finally reported with compelling extract examples. This method aligns with the interpretive approach as it allows for an in-depth exploration of the meanings embedded in participants' narratives.

Atlas.ti was used during the initial phases of the analysis to organize and establish preliminary codes, while subsequent phases were carried out manually. The data analysis process was iterative and reflexive, involving regular debriefing meetings to discuss and reach consensus on coding and theme development thus ensuring the credibility and trustworthiness of findings. Data saturation was determined through this ongoing process by assessing the recurrence of themes and the absence of significant new information. Reflexivity was maintained throughout the process, with researchers continuously reflecting on their own perspectives and influences [31].

Quotations from the participants' interviews were selected to illustrate the identified themes. To ensure methodological rigor, a qualitative methodology expert reviewed the findings. Additionally, member checking was conducted by sharing preliminary results with participants, who confirmed that the findings resonated with their perceptions and did not suggest major changes. This rigorous approach ensured the credibility and authenticity of the analysis [31].

### 2.6. Participant Demographics

A total of 19 participants were included in the study. Participants' ages ranged from 25 to 60 years, with varied years of experience and academic backgrounds. Most participants were female, reflecting the general demographic of the nursing profession in the region. Participants held diverse roles, including direct patient care nurses, outpatient clinic nurses, advance practice nurses, and nursing supervisors or coordinators. Regarding academic qualifications, participants ranged from bachelor's degrees to doctoral studies, with several holding postgraduate diplomas or master's degrees (see Table 2). Concerning clinical specialties, participants represented multiple fields including medical-surgical nursing, oncology, neurology, outpatient care, and chronic care management. It is important to note that some participants had experience across more than one specialty or work area, reflecting the multifaceted nature of nursing roles in hospital settings.

## 3. Results

Data analysis and response coding revealed the interaction of four themes and 13 subthemes (see Table 3). These themes and subthemes capture the perceptions of nurses and nursing managers regarding the implementation of VBHC and outcomes measurement in hospital settings.

### 3.1. Key Elements of Value in Healthcare

This theme focuses on the fundamental characteristics that define value in healthcare from a nursing perspective. The first important aspect identified by participants was optimizing time which addresses various aspects related to time management in healthcare. On the one hand, participants highlighted the need to reduce and minimize waiting times that people experience; on the other hand, optimizing times during the appointments was identified as essential to ensure adequate care. Participants emphasized the importance of dedicating sufficient time to each person to receive comprehensive and personalized attention. They also highlighted the need to provide efficient care by avoiding unnecessary delays, consolidating visits, and minimizing patient redirections between different areas or healthcare professionals.

*In hospitals, it is widely accepted that… “during the morning they [doctors] will come, in the morning, I will take care, during the morning… “During the morning? What does it mean during the morning? The patient's experience will be marked by this waiting time, and in my opinion, it is vital to start thinking in terms of patient experience (P5, Direct-care nurse)*

*For me, the value is no delays, that patients receive the care they need when they need it. (P3, Direct-care nurse)*

Another essential element of value is the use of PROMs, described as tools for aligning care with what truly matters to patients. Nurses noted that these instruments allow patients' concerns, goals, needs, and priorities to be objectively voiced, fostering clearer and more open communication between the person and the healthcare team. PROMs can/could help avoid unnecessary questions by providing information upfront, which makes consultations more efficient and allows healthcare professionals to focus on the most relevant health issues. Nurses emphasized that PROMs are especially useful for discussing sensitive topics that patients might otherwise hesitate to bring up.

*I think it is the way to give them a voice. (P1, Direct-care nurse)*

*The PROMs will also allow us to communicate and understand each other better without asking so much because patients will have already told us what matters most to them, right? and their needs…” (P5, Direct-care nurse)*

*“PROMS can improve communication and avoid communication errors, such as playing telephone” (P7, Direct-care nurse)*

Additionally, the implementation of PROMS is seen as empowering for patients, providing them with a sense of control over their care and health outcomes, increasing well-being and engagement in their treatment.

*I think that, first of all, it would bring control to the patients. And from this control, a greater sense of well-being could be derived, right?” (P8, Direct-care nurse)*

Participants noted that having self-reported data available before consultations helps streamline the process, reducing the need for repetitive questioning and allowing healthcare providers to focus on adapting care plans according to each individual's specific needs and priorities. Furthermore, nurses remark that analyzing self-reported results can help anticipate and avoid complications by identifying patterns or trends in the individual's health. The availability of this objective and quantifiable information reduces the likelihood of misinterpretations and helps avoid biases in data interpretation.

Importantly, participants stressed that PROMs must be meaningfully integrated into workflows, rather than collected as a mere formality. This points to a tension between transformative potential of PROMs and the risk of them becoming a bureaucratic task.

*Having this data will help us to make the visits more efficient and focused on what really matters. (P3, Direct-care nurse)*

*Of course, they are going to contribute (PROMs) if they are used well because it is not just that I am going to collect the data, but that I am going to collect them and I will be able to talk to the patient about what worries him the most (P2, Direct-care nurse)*

In addition, PREMs were considered crucial for enhancing quality of care. PREMs were seen as capturing aspects of the care experience that might otherwise remain invisible, serving both as a driver of continuous improvement and as a means of highlighting positive aspects of care.

*PREMS would also help to show in some way both the good and the bad. Complaints always come, but thanks are sometimes forgotten too, you know? And it would be a way of saying well, hey, we have strong points. The patient is very happy, he is very well attended here, but we must improve this (P9, Nursing manager)*

Taken together, PROMs and PREMs were seen as complementary instruments and nurses highlighted that using both is necessary to capture what patients consider most important. Participants consistently described their usefulness at the microlevel, where these measures improve the quality of patient–professional interactions and the tailoring of care to individual needs.

### 3.2. Conceptualization of Value-Based Care Through Established Healthcare Models

This theme explores how participants define and understand value in healthcare by drawing on conceptual parallels with established models. When asked to articulate what value means, participants often referred to concepts related to person-centered care (PCC). They emphasized the importance of focusing care on the individual, underscoring the need to personalize and tailor care to each patient's unique preferences, needs, and expectations. Nurses emphasized that aligning professional and patient perspectives avoids generalized approaches and ensures that goals and care strategies are tailored specifically to each person.

*“Not all of us have the same concerns or the same desires or the same dreams or want the same things and that also applies when we talk about what I expect from the health system. For me that is personalizing care and that is adding value.” (P2, Direct-care nurse)*

*“Everything that we consider valuable, sometimes it is not valuable for the patient… and vice versa, sometimes it is not so valuable for the patient.” (P6, Direct-care nurse)*

To provide valuable care, participants highlighted the importance of adopting a holistic and comprehensive approach to healthcare. One participant used the analogy of a puzzle, where each aspect of care represents an essential piece contributing to the patient's overall well-being.

*“It's as if each piece represents a different aspect of their health and well-being, from medical treatment, how they feel, physical aspects, to their emotional needs and personal preferences. The more precise and well-placed the pieces are, the clearer the overall picture will be of how we can provide quality, patient-centered care. The idea is that, to truly understand the value, we need to see the complete picture and not just the individual parts of the puzzle, because it will never be complete otherwise, and care will never be patient-centered if we do not listen to them explain the other pieces to us… (P1, Direct-care nurse)”*

Additionally, participants underscored the importance of actively involving the person in their own care process, moving away from the passive approach associated with the term “patient.” Participants emphasized the need to empower individuals to take an active role in managing their health, as they have a deeper understanding of their own conditions. Participants valued the personal perspective that each person brings to their care experience, recognizing the importance of understanding “value” from the point of view of those receiving care.

*“One of the things that caught my attention about this model is precisely that it includes the patient, who is truly at the center, as if they are driving, right? So, they need to be active from the start of their care, taking charge and saying: ‘Hey, you may know more about the disease, but I know about my life.'”(P1, Direct-care nurse)*

Participants also drew parallels with the concept of humanized care as a fundamental principle of value in healthcare. Providing empathetic and close care—where patients feel heard, accompanied, and understood—was considered essential for improving their experience. The participants reported that humanized care emphasizes empathy and the quality of human interaction, underscoring that while medical outcomes might remain the same, the experience can vary significantly depending on how patients are treated on a personal level.

*“There is value when we treat with closeness and empathy, because the patient's experience can vary greatly depending on the care they receive. They may heal in the same way, but certainly their experience will not be the same if there is value in capital letters in the care they receive compared to if there is not.” (P3, Direct-care nurse)*

Participants observed that while patients initially value the speed and efficiency of diagnostic results, over the course of their hospitalization, they tend to appreciate humanized aspects of care more. This includes the emotional support nurses provide, their efforts to make patients feel comfortable, and meaningful interactions that foster a sense of being genuinely understood. As one nurse noted, patients often remember the empathetic actions of their caregivers more than the technical aspects of treatment.

*“It seems that, initially, the value they place is more at the speed of diagnostic results, right? They want everything quickly, but then throughout the hospitalization, what they end up valuing more is that closeness, that humanization—not just having been with them or how you calmed their child, for example, without having to force them to undergo a procedure. They remember that more, right?” (P11, Direct-care nurse)*

The last parallel identified by participants in defining value in healthcare is shared decision-making. Along with patient empowerment, shared decision-making was recognized as essential for valuable healthcare delivery. This approach promotes a collaborative process where decisions are made as a team, involving both the healthcare team and the patient. However, participants also emphasized the importance of respecting the patient's preferences regarding their level of participation, incorporating them to the extent they wish.

*“I think it's essential to include patients in the conversation. Not just to collect data for the sake of it, but to really understand what they expect from us. If we are talking about quality of life, for example, how can we, as a team, respond to that?” (P4, Direct-care nurse).*

*“I would gauge the type of patient and see to what extent they want to be involved in this or not, because I've encountered all sorts of cases, right? (…) But I would say, rather than defining the roles of the patient and myself, I would leave it a bit more open and say that it should be the patient who decides to what degree they want to participate. Obviously, keeping in mind that the more initiative-taking they can be, the better.” (P6, Direct-care nurse)*

Nurses observed a shift in expectations among some patients, particularly younger ones, who increasingly view participation and value not as optional but as fundamental expectations.

*“I believe that younger patients come in a different way; it's like a generation that already has so much information and misinformation that they start speaking this language of PROMs without even realizing it… They come in and directly show you what worries them and what they want, you know?” (P4, Direct-care nurse)*

Nurses conceptualized value primarily through the lens of microlevel interactions—personalization, humanization, and collaboration with patients. At the same time, they linked these ideas to broader mesolevel professional norms, such as team practices around shared decision-making, suggesting that their understandings are shaped by both individual encounters and institutional discourses promoting person-centeredness.

### 3.3. Practical Aspects of Implementing VBHC

This theme addresses the strategies and practical aspects necessary for implementing VBHC. It encompasses practical steps for effective integration, highlights key facilitators that support adoption, and identifies potential barriers that may hinder successful implementation. Central to their accounts was the need for a clear and shared strategy to guide the transition towards a value-based care model. This includes interdisciplinary collaboration ensuring team consensus and active participation of all members. For nurses, agreeing on common criteria and integrating PROMs into clinical workflows were seen as essential to prevent their use from becoming fragmented or superficial.

*“Of course, there needs to be a clear strategy, right? A plan to follow; otherwise, it is just an idea or a trend, but without a plan and without working together, it remains a cool initiative that does not move forward….” (P1, Direct-care nurse)*

*“But in the end, it's about everyone receiving the same attention. If I am the one seeing the PROMs, whether it is my substitute or my colleague… it is necessary to unify criteria so that we all follow the same line regarding the PROMs report we have in the records, you know? We need a collective approach.” (P7, Direct-care nurse)*

As a practical action, participants suggested adapting and diversifying patient-reported data collection methods to align with the patients' skills and needs. Nurses also emphasized the importance of establishing an efficient operational framework for data collection and visualization, ensuring that the latter is intuitive and accessible, and does not require navigating through multiple screens or steps. Nurses believe that creating a clear and user-friendly visual experience is the key for all users.

*“The way to collect and present the data will be fundamental. It absolutely has to be easy to understand and straightforward to complete for patients, and it should not be just another mountain of clicks and steps in SAP for us. It needs to be easy to see how those PROMs evolve clearly and without complications, we need to think it through and design it that way.” (P15, Direct-care nurse)*

*“There's a lot of diversity because we have people who would respond through an app, which would be great for us. But there are others who would need a phone call, and some who might prefer an email due to their work situation. I think we should have various tools to reach everyone because each person, each patient, is a world. We need to adapt to their needs.” (P12, Nursing manager)*

Participants also underscored the need for training healthcare personnel and raising awareness about the overall VBHC model and its function. They emphasized that it is crucial for both professionals and the community to understand the importance of VBHC, ensuring that everyone recognizes it as an integral part of healthcare delivery and believes in its significance. Regarding PROMs, participants highlighted the importance of helping patients understand how these tools contribute to personalizing their care, ensuring they perceive a tangible benefit from their use.

*“The right training, support within the team, and open communication are key elements. We also need a shared understanding of the benefits so that everyone is on board.” (P4, Direct-care nurse)*

*“You know, what I used to explain it on my own had little support, let's say. I didn't get much response, and it didn't seem like the patients believed in it much, I don't know, but now that it's coming from the team, that we all explain it, and the patients see its importance…” (P14, Direct-care nurse)*

Participants identified several enablers that promote the effective implementation of VBHC. They highlighted the importance of motivated and engaged teams, as well as a shared perception of VBHC's benefits among healthcare professionals. Nurses noted that aligning all healthcare providers with this perspective could significantly facilitate the adoption of VBHC. Drawing on successful experiences and external benchmarks was also mentioned as a key facilitator for implementation. Learning from other institutions that have successfully implemented VBHC can provide valuable insights and guidelines. Similarly, participants identified internal diffusion effects as another facilitator, which can boost implementation and enable gradual propagation across different settings. Additionally, participants valued the presence of mentor figures to guide the team during implementation, sharing expertise and offering encouragement.

*“Young and motivated teams can be a great boost for implementation.” (P14, Direct-care nurse)*

*“Raising awareness among healthcare and non-healthcare professionals about the usefulness of what has been worked on, what has been achieved in other places, showing results. When we present the outcomes from other places, like ‘look, this is used in this location for this purpose, and they've seen these benefits, they've improved this'…” (P9, Nursing manager)*

*“If there were a reference person like there is for other things, we could have that, and it might be easier to implement because this person would always be there as a guide…” (P13, Nursing manager)*

In contrast, nurses pointed to significant barriers. These included systemic constraints such as limited time and resources, including financial support, personnel, and time. They highlighted that an overburdened healthcare system leads to exhausted professionals, making it challenging to adopt additional approaches like VBHC.

*“I believe that as a center, we still lack many healthcare professionals, and we don't always reach everywhere. Many times, we get there because we put in a lot of personal effort and time…” (P15, Direct-care nurse)*

*“Yes, that's a barrier, being burned out or demotivated… Well, that and the pressure of time.” (P17, Direct-care nurse)*

In this sense, time constraints present an additional barrier to the implementation of PROMs, as participants suggested that these tools are likely to be deprioritized when there are multiple demands and responsibilities. In such situations, PROMS may be relegated to a secondary position in favor of other tasks considered more urgent.

*“At the end, the PROMs won't be used if there's no time because we'll prioritize other things…” (P1, Direct-care nurse)*

Another barrier identified is the digital/generational divide, which could particularly affect the collection of self-reported data. Participants expressed concern about how older patients might struggle with the technology required to complete and visualize patient-reported data. Resistance to change among professionals further reinforced these challenges, and this reluctance to adopt new methods can hinder the implementation of tools like PROMs and limit the broader adoption of VBHC practices.

*Yes, it is true that just as I told you, there is the threat of people who say that things have always been done this way (P16, Direct-care nurse).*

Lastly, participants pointed to organizational culture and volume orientation as significant barriers within healthcare systems. They observed that prioritizing quantity over quality in care hinders the adoption of person-centered approaches like VBHC.

*“We are oriented towards quantity, let's say, and we need to make a cultural shift towards quality.” (P12, Nursing manager)*

Overall, nurses framed implementation as a process that depends less on technical availability than on organizational readiness and cultural alignment. Their accounts suggest that the sustainability of VBHC requires not only tools and guidelines but also investment in collective motivation, adequate resourcing, and a shift in professional and organizational practices.

### 3.4. Opportunities and Systemic Challenges in Value-Based Healthcare

This theme explores the broader implications of the VBHC model, focusing on how it will impact the role of nurses, patient experience, and the structure of the healthcare system. Unlike the practical aspects of implementation, which address concrete actions and immediate challenges, this theme delves into nurses' perspectives on the future of their profession within VBHC, reflecting on the systemic and structural challenges that may affect its sustainability and alignment with patient-centered care.

Nurses expressed optimism about their role in VBHC, seeing it as an opportunity for greater recognition and empowerment within the profession. They believe that the professional profile and skills of nurses are particularly suited to play a significant role in this care model, where nursing is seen as crucial not only in direct care but also in coordinating comprehensive care. This perspective emphasizes enhanced autonomy and responsibility for patient management. VBHC is viewed as an opportunity for nurses to assume leadership roles, increasing their responsibilities in clinical decision-making and care coordination.

*“Nursing should be the force that connects theory with practice. In other words, we don't just implement; we lead the change.” (P7)*

*“Nursing must be a fundamental pillar, not only as executors but as the real connection between the patient and the healthcare system.” (P4)*

At the same time, participants expressed concerns about the risks associated with implementing PRMs within this framework. They feared that data collection might become an additional workload, shifting their role towards administrative tasks and distancing them from patient care. They also worried that patients might experience PRMs as an additional burden, and that the model could raise unrealistic expectations, leading to frustration if these were not met. Moreover, a recurrent concern was that VBHC might be seen as a passing trend rather than a sustainable improvement.

*“I hope it's not just a trend that comes and goes. I have seen many of those; we get motivated, form working groups, and then when we have it, they change directions and objectives, and it is on to the next thing. I really hope this is a commitment for the present, but with a future.” (P1)*

In relation to broader systemic issues, participants expressed skepticism and doubt about the implementation and long-term effectiveness of VBHC. Many participants observed that certain fundamental problems in the healthcare system need to be addressed before implementing additional changes, raising doubts about the system's readiness to adopt a value-based care approach. Participants also questioned the feasibility of applying PRMs equally in inpatient and outpatient settings. While its utility in outpatient consultations and follow-up is recognized, there is lingering uncertainty about whether PRMs are equally applicable in both settings.

*“I find it hard to believe that this is going to work well if there are structural things that aren't resolved, I find it difficult to propose advancing towards a new model of care like this…” (P17)*

*“We come here with much more advanced ideas when we see foundational issues that are still not fixed, and that's frustrating because in the end, if they want to do big things, the foundations still aren't set.” (P3)*

Furthermore, participants highlighted a perceived misalignment between the theoretical discourse around value-based care and its practical implementation. Although the rhetoric emphasizes patient-centered care, some nurses observed that daily practices often prioritize organizational needs over patient value. This perceived discrepancy generates skepticism among nurses about the system's ability to consistently deliver value-based care.

*“Sometimes I feel like we're swimming in a discourse very focused on the patient, but we're drowning… We talk about value, but in day-to-day reality, value means approving (care plan) agendas, providing basic care, sticking to schedules, and mind you, making sure the bed is free by whenever… I do not doubt the intention, but I don't see much alignment with the value we're talking about…” (P11).*

In summary, nurses framed VBHC as a double-edged process: it not only offers professional growth and recognition but it also raises concerns about patient burden, sustainability, and systemic preparedness. Their accounts reflect not only optimism about nursing's role but also skepticism about whether broader organizational and systemic conditions will allow VBHC to fulfill its promise.

**Figure 1:**
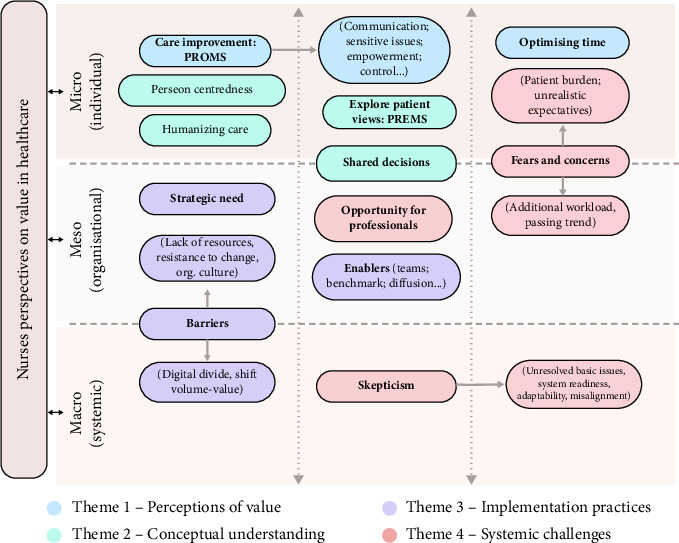
Conceptual synthesis of results: nursing perspectives on value in healthcare across micro-, meso-, and macrolevels. Subthemes are shown in bold, with illustrative codes in smaller font where needed to clarify meaning.

Beyond presenting each theme in detail, it was deemed important to provide a visual synthesis of the findings. To this end, a conceptual diagram was developed to illustrate how the four themes and 13 subthemes (Table 3) can be understood across microlevel (individual), mesolevel (organizational), and macrolevel (systemic), offering an overarching view of nursing perspectives on value in healthcare (Figure 1).

## 4. Discussion

### 4.1. Key Elements of Value in Healthcare

Time optimization emerged as a key determinant of value in healthcare, encompassing factors such as waiting times, delays, and time to obtain results. Participants emphasized timely access to care, consistent with Beattie's model of care quality, which highlights timely care delivery as fundamental to PCC [33]. Reducing waiting times and optimizing consultation durations have been recognized as essential to improving patient satisfaction [34], and the NHS patient experience framework further underscores the role of time as a key dimension influencing patient experience [35]. These findings are consistent with previous research that highlights the dual benefits of reducing waiting times: to enhance patient satisfaction and improve operational efficiency and resource utilization in healthcare settings [36]. Addressing these time-related challenges enables healthcare providers to meet both patient needs and institutional goals, thereby aligning with principles of VBHC.

Participants also identified PROMs as essential to enhancing personalized care, improving communication, and facilitating data-driven care. This finding aligns with literature underscoring the role of PROMs in improving patient-provider communication, avoiding misinterpretations [37, 38], and increasing patient involvement in their own care [39]. By collecting PROs prior to consultations, healthcare teams can address pressing concerns more efficiently. Similarly, Sassen [40] underscores the utility of PROMs in VBHC, while Aiyegbusi et al. [41] note that electronic PROMs are increasingly accepted in clinical practice and positively impacting patient-centered outcomes.

PROMs also play a crucial role in addressing aspects of health-related quality of life (HRQoL) that might be overlooked. Greenhalgh et al. [42] note that PROMs can influence how patients reflect on their health and prompt discussions about issues that patients might not typically raise, a point echoed by participants, who suggested that PROMs provide a platform for addressing difficult-to-discuss topics. Recent studies reinforce the benefits of PRMs. Chung et al. [43] found that patients appreciate regular consultations aimed at addressing unmet needs, fostering trust with care teams. Similarly, Consolo et al. [44] observed that electronic PROMs are effective tools for monitoring symptoms and expressing concerns about less explored aspects of care in palliative context. However, the current study, in line with Thestrup Hansen et al. [45], highlighted that PROMs only deliver value if they are actively used in practice. Greenhalgh et al. [42] also caution that while PROMs can help patients voice concerns, they do not always change clinician communication practices. To fully realize the benefits of PROMs, clinicians must integrate them into a broader understanding of patient care.

Litchfield et al. [46] raise concerns about the efficacy of PROMs, with some general practitioners questioning their advantages over traditional consultations. Nurses in our study primarily focused on the individual-level benefits of PROMs, emphasizing their role in improving patient–provider communication and personalizing care. However, they did not explicitly discuss the potential population-level applications of these tools. Interestingly, Daniels et al. [47] highlight that PROMs can be valuable beyond individual use, serving as tools for benchmarking and continuous improvement and offering system-level benefits to enhance quality and efficiency in healthcare organizations.

Participants emphasized the importance of PREMs as the third key element of value, particularly for their ability to capture aspects of patient experience often overlooked by clinicians. PREMs provide nurses with deeper insights into patients' experiences, enabling subtle yet meaningful improvements in care delivery. This finding aligns with De Rosis et al. [48] who found that systematic use of PREMs in healthcare institutions facilitated continuous quality improvement. While PREMs are generally seen as valuable, their routing use in practice remains debated, with some staff finding the information invaluable and motivating, while others were less certain about their usefulness [49].

Finally, the present study also highlighted that PREMs provide a comprehensive and balanced perspective of patient experience by identifying both areas for improvement and positive aspects of care. Shunmuga Sundaram et al. [49] note that positive feedback through PREMs can reinforce good practices and serve as a motivational tool for staff. Murante et al. [50] underscore the importance of feedback loops in enhancing patient satisfaction, showing that when clinicians and hospital staff are informed about survey results, they are better equipped to focus on areas needing improvement. Taken together, these findings support the argument that the implementation of PREMs, when combined with effective feedback mechanisms, can lead to significant improvements in both the quality of care and patient satisfaction.

### 4.2. Conceptualization of Value-Based Care Through Established Healthcare Models

Nurses in this study equated value with the principles of PCC, focusing on treating patients holistically and ensuring care reflects their preferences, emotional needs, and life circumstances. This approach requires aligning professional perspectives with those of the patient, so that strategies reflect what matters most to them. While PCC and VBHC share the goal of improving patient outcomes, they differ in emphasis and measures of success [51]. Tseng & Hicks [51] note that in VBHC, patient centeredness is only one quality aspect, whereas PCC focuses more broadly on patient and family preferences. Aligning these two perspectives appears essential to deliver care that is both responsive to individual needs and sustainable in terms of quality and cost-effectiveness.

Participants in this study advocated for centering and individualizing care, aligning closely with the goals of VBHC and the principles of PCC. As highlighted by the Picker Institute, these principles involve respecting patients' values and preferences ensuring integrated care, clear communication, providing emotional support, and engaging family and friends [52]. Nurses emphasized the importance of tailoring care to individual patient needs and preferences, avoiding generalizations and ensuring that patients feel seen and heard as individuals, key tenets of PCC. While our findings suggest that shared decision-making aligns with VBHC, Stephens et al. [53] offer a nuanced view, arguing that shared decision-making is not just a parallel concept, but rather a natural consequence of value-based interventions, and thus a potential area for further research and practice integration.

A strong parallel was also observed between VBHC and shared decision-making. Nurses emphasized that involving patients in their care decisions is crucial to empowering them and ensuring care aligns with their personal values. This finding is consistent with Sassen [40] and Havana et al. [54], who underscore the importance of involving patients in every aspect of care, not only considering medical options but also respecting their unique preferences and experiences. However, our findings, consistent with Havana et al. [54], indicate that not all patients prefer an active role in decision-making. Some opt for a more passive role, highlighting the need for flexible approaches to patient involvement in healthcare. The authors in [55] add that instances where patients are not interested in participating actively in their care may be attributed to a lack of understanding on how to engage them effectively. Therefore, healthcare providers must adopt pedagogical strategies to better meet individual needs.

Another key parallel drawn by participants was the notion of humanized care, where empathy and connection enhance the patient experience. Nurses observed that patients often remember compassionate interactions more than technical details, aligning with Kitson et al. [56], who identified that while technical competence is important, relational aspects of care are crucial for overall patient experience. This emphasis on empathy reflects the importance of recognizing the individual behind the patient and fostering meaningful connections between patients and healthcare providers, an idea also supported by Galvin et al. [57].

While participants primarily framed value at the microlevel, through personalization and humanized interactions, their emphasis on shared decision-making also resonates at the mesolevel. This practice requires team coordination, institutional endorsement, and professional norms that support collaborative care, suggesting that nurses' views are shaped not only by individual encounters but also by organizational discourses promoting participation and patient involvement.

The first two themes, which are more theoretical, reveal differences between the comprehensive definition of VBHC and nurses' perceptions. While VBHC is defined to encompass personal, technical, allocative, and societal value [2, 3], our findings suggest that nurses primarily focus on personal value when conceptualizing healthcare value. Participants consistently emphasized patient preferences, needs, and expectations, aligning closely with this personal value dimension of VBHC. There was also some recognition of allocative value, as nurses advocated for individualized patient attention and time. However, notably absent from most accounts were explicit references to cost variables or societal value. The strong focus on personal value reflects nursing's commitment to individualized, empathetic care yet may risk underrepresenting systemic and economic dimensions essential for the successful and sustainable adoption of VBHC. This result aligns with previous research highlighting that clinicians often perceive VBHC as an opportunity to focus more on outcomes while placing less emphasis on cost containment [58]. A recent concept analysis further underscores this fragmentation, emphasizing that the way value is defined in healthcare continues to be shaped by tensions between patient-centered care, quality of care, and economic factors [4]. Expanding leadership and targeted training to encompass all VBHC dimensions is therefore essential to bridge this conceptual divide and enable nurses to contribute effectively to system-wide transformation.

### 4.3. Practical Aspects of Implementing VBHC

Participants emphasized the critical need for a clear and unified strategy in implementing VBHC. Without this, VBHC risks become an abstract concept with little practical impact. This aligns with Thestrup Hansen et al. [59] and Cossio et al. [60], who identified the lack of strategy as one of the practical barriers to VBHC implementation. Similarly, Fernández-Salido et al. [61] highlight the importance of standardizing outcome measures, ensuring data accessibility and providing sufficient resources for successful implementation.

Interdisciplinary collaboration and standardized guidelines emerged as essential for consistent VBHC implementation. Coordinated efforts to integrate PRMs efficiently into practice were seen as crucial, reflecting Cossio et al. [60] who emphasize the role of multidisciplinary teams in achieving value-based care. Nurses underscored the importance of team consensus and participation, noting that the involvement of all healthcare professionals, not just specific departments, is vital for success.

Participants noted that training and sensitization were also keys to implementation. Eilayyan et al. [62] point out that healthcare providers often lack the necessary skills and understanding of how to implement and interpret value-based care initiatives. Li et al. [63] further support this, identifying that a significant percentage of Chinese nurses had limited knowledge about value-based care. Pittman et al. [64] add that a lack of educational preparation has limited the nursing profession's involvement in discussions around value-based payments and system change. Participants stressed that shared understanding of VBHC goals is crucial and achievable through comprehensive training programs and efforts to raise awareness among all stakeholders, addressing the concerns raised by Cossio et al. [60] regarding the lack of social acceptance of the VBHC concept within medical teams. The nurses' perspective underscores the need for leadership and training to broaden understanding of VBHC's multidimensional nature, empowering nurses to advocate for comprehensive value-based approaches.

To address these training needs, Eilayyan et al. [62] suggest a multicomponent approach, including workshops, educational materials, and regular use of score reports by healthcare professionals, while Daniels et al. [47] emphasize the need for practical knowledge beyond theory. Nurses echoed the need for practical training and tools for effective implementation.

Several key facilitators for VBHC implementation were identified. Motivated teams were seen as crucial, with participants noting that enthusiasm and commitment drive adoption. This finding is consistent with [65], who highlight the role of “champions” in successful implementation of new practices in healthcare settings.

Another powerful facilitator is the internal diffusion of positive outcomes, where sharing success stories and tangible benefits helps to foster broader acceptance within the organization. This idea aligns with Rogers [66], whose diffusion of innovations theory posits that the spread of new ideas is accelerated when peers communicate their positive experiences. Greenhalgh et al. [67] describe this phenomenon as “social contagion” in the context of innovation diffusion in healthcare organizations. Similarly, external benchmarks, where other institutions demonstrate the benefits of VBHC, were seen as valuable learning tools for motivating teams, echoing Greenhalgh et al.'s [67] observations on learning from external examples and adapting them to local contexts.

The role of mentor figures in guiding and supporting the implementation process was also emphasized, aligning with [47] emphasis on inspirational leadership to drive change. Participants highlighted the importance of mentors in providing support and navigating challenges.

Despite these facilitators, significant barriers to VBHC implementation were identified. The most pressing challenges included lack of resources, staffing shortages, and time constraints, which limit the ability of healthcare providers to fully embrace the VBHC model. These findings mirror broader concerns in the literature [18, 61], where inadequate resources, insufficient infrastructure, and support hinder VBHC success. Addressing these challenges may benefit from recent nursing workforce policy innovations, such as Park's [10] sweet-spot theory-driven implementation strategy, which emphasizes engaging all stakeholders to achieve an optimal balance between care quality, nurse staffing, and costs. This approach fosters the creation of sustainable shared value and offers a practical framework for applying value-based care principles within nursing management.

Organizational culture remains another notable barrier, where the focus continues to favor volume over value. Participants noted that many healthcare systems still measure success by the number of patients seen rather than by the quality of care provided, aligning with Cossio et al. [60] who point out that shifting from volume-driven to value-driven models requires a cultural transformation. Similarly, departmental structures and pay-for-volume contracts further impede this transition [47]. Resistance to change, often encapsulated in a “we've always done it this way” mindset, also emerged as a major obstacle and has been identified by Wendt et al. [68] as a key factor sustaining low-value practices in healthcare delivery.

Lastly, participants pointed out the digital and generational divide as a barrier to VBHC implementation. Meirte et al. [69] raise concerns about the exclusion of certain patient populations, particularly older adults, and those with limited access to technology, who may struggle to engage with the digital health tools. This digital divide poses a risk of leaving vulnerable populations behind in VBHC initiatives.

### 4.4. Opportunities and Systemic Challenges in Value-Based Healthcare

Nurses consistently view VBHC as an opportunity for greater visibility and empowerment within the healthcare system, aligning with research that underscores nurses' leadership potential in implementing value-based care initiatives [70, 71]. The holistic approach, coordination skills, and close patient relationships position nurses as ideal VBHC leaders. Reference [15] demonstrated the effectiveness of nurse-led models in improving health outcomes, while Johnson et al. [72] note their potential to address social determinants of health and advance VBHC principles, supporting the notion that nurses are well-suited to lead VBHC initiatives. Similarly, Paulus and Kurosaka [73] emphasize the crucial role of nursing leadership in driving the transition to value-based care models, noting its importance in advancing care and achieving better patient outcomes.

The inherent qualities of the nursing profession—empathy, patient-centeredness, and holistic support—make nurses particularly suitable for VBHC implementation. Rodríguez-Pérez et al. [74] describe nurses' values of humanity, compassion, and respect, which position them well to lead VBHC initiatives and improve both patient satisfaction and clinical outcomes. By leveraging these intrinsic qualities, nurses are well positioned to implement and advocate for VBHC principles effectively. In this line, Pittman et al. [64] emphasize that empowering nurses through training and leadership opportunities is the key to engaging them in healthcare system redesign and value-based models.

While nurses are well-positioned to lead VBHC initiatives, participants note that successful implementation requires multidisciplinary collaboration. Daniels et al. [47] suggest that including all necessary disciplines in Value-Based Quality Improvement (VBQI) teams is critical to ensure shared responsibility and motivation.

Nurses are also seen as ideal candidates for implementing PROMs. Their close interactions with patients give them unique insights that are essential for the success of VBHC, especially in the implementation of PROMs. Arora & Haj [75] and van Egdom et al. [76] demonstrate that specialist nurses play a significant role in managing patient outcomes and leading the entire process of PRO implementation.

Despite the opportunities, concerns persist regarding VBHC's long-term impact. One significant worry is that VBHC may become a passing trend, a common fear in healthcare systems, as highlighted by Thestrup Hansen et al. [45]. This concern underscores the need for sustained institutional support and clear long-term strategies for VBHC implementation, leading to frustration among staff and unmet goals. Additionally, increased workload, particularly with administrative tasks, was another major concern. Graupner et al. [77] and Cossio et al. [60] note that the administrative burden of VBHC can overwhelm healthcare providers, especially when formal training programs and clear guidelines are lacking.

Finally, there is concern about the potential for unrealistic patient expectations. Aiyegbusi et al. [78] highlight that while patients are generally willing to engage with VBHC initiatives like electronic PROMs, clinicians worry that these efforts might create expectations beyond what healthcare systems can deliver. This disconnect underscores the importance of managing expectations to avoid patient dissatisfaction and mitigate legal risks.

Systemic skepticism also emerged, with participants questioning whether the healthcare system is truly ready for VBHC. Unresolved issues such as staffing shortages and inadequate infrastructure were highlighted, reflecting concerns in the literature [61] that standardized outcome measures and proper resource allocation are critical for successful VBHC implementation. Participants also expressed uncertainty about VBHC's adaptability across various healthcare contexts. While its potential in chronic and outpatient care was acknowledged, its applicability in settings like emergency care remains unclear. Cossio et al. [60] point out that without a cohesive framework and clear benchmarks, VBHC risks being inconsistently applied across healthcare settings, further complicating its implementation.

A key issue raised by participants was the discrepancy between the theoretical ideals of VBHC and its practical application. Several participants pointed out that healthcare decisions are often driven by organizational priorities rather than patient-centered principles. This misalignment between rhetoric and day-to-day actions is not uncommon in healthcare systems, where “value” is frequently equated with cost reduction, quality improvement, or efficiency metrics, rather than genuine improvements in patient outcomes [79]. This observed gap between VBHC ideals and real-world implementation can be further elucidated by adopting a multilevel perspective. Mattia et al. [80] argue that effective VBHC adoption requires alignment and coherence across individual, organizational, and systemic levels. These concerns reflect macrolevel challenges, where unresolved structural issues and systemic priorities may undermine the sustainability of VBHC despite positive experiences at the micro- and mesolevels.

Taken together, our findings highlight how nurses articulate value across interconnected levels: microlevel care interactions, mesolevel organizational practices, and macrolevel system structures. Recognizing these interdependencies is the key to understanding both the opportunities and the tensions of VBHC implementation in real-world contexts.

### 4.5. Limitations

This study has some limitations that must be considered. First, the sample was limited to nurses and nursing managers from tertiary public hospitals in Barcelona, Spain, which may limit the applicability of the findings to other healthcare settings or regions. Additionally, the study's focus on the nursing perspective means that insights from other healthcare professionals, such as physicians or administrators, were not explored, which may affect the broader applicability of the findings in interdisciplinary VBHC implementation. The sample was also predominantly female (16 of 19 participants), reflecting the gender distribution of the nursing workforce in the region, as reported in previous studies, and may limit the exploration of perspectives from male nurses. Moreover, although a formal audit trail was not maintained, methodological rigor was ensured through collaborative analysis and regular consensus meetings among researchers. Member checking was also conducted, validating findings and enhancing credibility. Potential social desirability bias should also be acknowledged, particularly given the generally positive framing of PRMs expressed by participants. Finally, although this research explores how VBHC could impact patient care, direct patient experiences and perspectives were not included and should be further examined in future studies.

## 5. Conclusion

The integration of VBHC into nursing practice presents both substantial opportunities and formidable challenges. While nurses emphasize personal and allocative value in their understanding of VBHC, there is a notable absence of cost considerations and societal value in their definitions. This gap suggests a limited understanding of the comprehensive VBHC framework, which could hinder its comprehensive implementation.

The current findings underscore that the importance of clear strategies, interdisciplinary collaboration, comprehensive training, and strong leadership are keys to overcoming implementation barriers. Key facilitators include leveraging external benchmarks, promoting the internal diffusion of successful practices, and providing mentorship. PROMs and PREMs remain valuable tools for enhancing patient-centered care, but their success depends on well-coordinated, resource-supported efforts.

For nurses, VBHC represents a significant opportunity to take on a more active role within the healthcare system, focusing on patient-centered care. The nursing profession is well-positioned to contribute to VBHC initiatives by leveraging their unique skills, patient-centered approach, and ability to collaborate across disciplines. By embracing these opportunities and addressing the identified gaps, nurses can enhance their professional recognition and make meaningful contributions to improving healthcare outcomes and value.

## 6. Implications for Nursing Management

1. Educational initiatives: Prioritize the development of comprehensive educational programs that encompass all dimensions of VBHC—personal, technical, allocative, and societal. Develop and integrate targeted training modules on aspects related to VBHC (such as PROMs) within continuous professional development (CPD) programs. This will equip nurses with a more holistic understanding of value in healthcare and provide them with the skills needed to implement it effectively.
2. Cultural shift: Lead efforts to shift organizational culture from volume-based to value-based care, emphasizing long-term patient outcomes over short-term metrics. This could include embedding PROMs feedback into daily clinical workflows such as EHR and routine team sessions to ensure outcomes drive care decisions.
3. Interdisciplinary collaboration: Foster environments that encourage and facilitate collaboration across different healthcare disciplines to ensure a unified approach to VBHC implementation.
4. Leadership development: Identify and nurture nursing leaders who can champion VBHC initiatives, providing them with the necessary support and resources to drive change. Encourage the identification and support of nurse “champions” on each unit who advocate for VBHC.
5. Continuous evaluation: Implement systems for ongoing assessment of VBHC initiatives, using both quantitative (e.g., PROMs scores) and qualitative measures to monitor their impact on patient outcomes and overall healthcare value.

By focusing on these areas and concrete strategies, nursing management can play a crucial role in overcoming the identified barriers and realizing the full potential of VBHC in improving healthcare delivery and patient outcomes.

## Figures and Tables

**Table 1:** Interview guide.

Semistructured interview guide
Position/academic background: Age: Gender: Date and time:

• What do you understand by value in healthcare? How do you define value-based care? • How do you believe PROMs or PREMs can contribute to the journey towards value-based care? • If applicable, could you share your experience with PROMs or PREMs from a nursing perspective? • Can you think of any examples from your regular practice where integrated PROMs would/or have been useful? • What potential do you see in your regular practice/area of expertise for PROMs? • What role do you think nursing can play in the implementation of this value-based care model? • Regarding the patient, what do you think should be their role from this new perspective? • From the user's perspective, what do you think this new approach would bring to the people you care for/support? • What barriers do you encounter currently in steering care towards value? • And what are the facilitators? • Regarding the collection of PROMs and PREMs, what barriers do you think might hinder this? • Do you believe your department/hospital/unit is prepared for the implementation of value-based care? Please explain. • How do you think it impacts or how would you like it to impact their care in the future? • What do you think is needed for this new approach to succeed? • Is there anything you would like to add that I haven't asked you?

**Table 2:** Participant demographics.

Sample description	*N* participants
Professional role	
Direct patient care nurses on hospital wards	8
Nurses in outpatient clinics	4
Advanced practice nurses	3
Nursing supervisors and coordinators with nursing background	4
Age	
18–25	2
26–40	11
41–65	6
Sex	
Man	3
Woman	16
Others	0
Academic level	
Bachelor's degree	1
Postgraduate diploma	8
Master's degree	9
Doctorate	1

**Table 3:** Thematic analysis results.

Theme	Subthemes	Codes
Key elements of value in healthcare	Optimizing time	Reducing waiting times Optimizing attention time
	Use of PROMs for care improvement	Needs and priorities Improved communication Sensitive issues Sense of control Data available prior to consultations Efficient care Anticipate and avoid complications Avoid misinterpretations Not just measuring
	Exploring patient views through PREMs to enhance care quality	Capture unnoticed aspects Driver of improvement Highlight the positive

Conceptualization of VBHC through established healthcare models	Person-centered care principles reflect core elements of value	Individualizing and personalizing Preferences Individual needs Expectations Aligning the professional-patient vision Holistic and comprehensive approach Active patient engagement Patient perspective on value
	Humanizing care as a reflection of valuable nursing practices	Empathy Closeness Making patients feel heard Accompanying
	Shared decision-making as a model for valued collaborative care	Making decisions as a team Patient empowerment Preferences of involvement Shift in expectations

Practical aspects of implementing value-based healthcare	Strategic action steps	Clear strategy Team consensus Team member participation Interdisciplinary collaboration Standardizing action guidelines Diversity in data collection Training personnel Sensitizing everyone Efficient operational framework
	Success enablers	Motivated teams Perception of benefits/utility External benchmarks Internal diffusion Identifying mentor figures
	Barriers to implementation	Lack of resources Digital/generational divide Resistance to change Organizational culture Volume orientation

Opportunities and systemic challenges in value-based healthcare	Opportunities for professional growth	Recognition Nursing role empowerment Suitable nursing profile
	Fears and concerns	Passing trend Additional workload Shift towards administrative tasks Patient burden Unrealistic expectations
	Systemic skepticism	Unresolved basic issues System readiness Adaptability to different contexts Misalignment between theory and practice

## Data Availability

The narrative data generated and analyzed during the current study are not publicly available due to ethical and privacy restrictions.
